# Helpful Female Subordinate Cichlids Are More Likely to Reproduce

**DOI:** 10.1371/journal.pone.0005458

**Published:** 2009-05-06

**Authors:** Dik Heg, Eva Jutzeler, Jeremy S. Mitchell, Ian M. Hamilton

**Affiliations:** 1 Department of Behavioural Ecology, Institute of Ecology and Evolution, University of Bern, Hinterkappelen, Switzerland; 2 Department of Evolution, Ecology and Organismal Biology, Department of Mathematics, The Ohio State University, Columbus, Ohio, United States of America; University of Edinburgh, United Kingdom

## Abstract

**Background:**

In many cooperatively breeding vertebrates, subordinates assist a dominant pair to raise the dominants' offspring. Previously, it has been suggested that subordinates may help in payment for continued residency on the territory (the ‘pay-to-stay hypothesis’), but payment might also be reciprocated or might allow subordinates access to reproductive opportunities.

**Methodology/Principal Findings:**

We measured dominant and subordinate female alloparental brood care and reproductive success in four separate experiments and show that unrelated female dominant and subordinate cichlid fish care for each other's broods (alloparental brood care), but that there is no evidence for reciprocal ‘altruism’ (no correlation between alloparental care received and given). Instead, subordinate females appear to pay with alloparental care for own direct reproduction.

**Conclusions/Significance:**

Our results suggest subordinate females pay with alloparental care to ensure access to the breeding substrate and thereby increase their opportunities to lay their own clutches. Subordinates' eggs are laid, on average, five days after the dominant female has produced her first brood. We suggest that immediate reproductive benefits need to be considered in tests of the pay-to-stay hypothesis.

## Introduction

Subordinate individuals in group-living vertebrates may assist a dominant breeder pair by helping to raise the dominants' offspring [1]–[3]. In many cases, subordinates may gain kin-selected benefits by doing so because the subordinates are related to the dominant pair [4]. However, genealogy reconstructions using molecular markers have shown that subordinates are often not related to the recipients of the subordinates' helping behaviour [5]–[10]. In these cases, helping behaviour cannot be attributed to kin-selected benefits and, therefore, subordinates are expected to gain other benefits. Such benefits might include establishment of a work force that will be present already when the subordinate inherits the dominant breeding position [11]–[13], being allowed to stay in the group (‘pay-to-stay’) and receive survival benefits [8], [14]–[19], or access to breeding resources for the subordinate's own reproduction [20], [21].

Previously, the ‘pay-to-stay’ hypothesis has been invoked to explain why unrelated subordinates show helping behaviour in the social cichlid *Neolamprologus pulcher* (see references above and review [22]). Under this hypothesis, subordinates pay by helping in exchange for acceptance in the group acceptance in the group. Group membership confers access to group-held resources and increases a subordinate's likelihood of surviving long enough to obtain a breeding position in the future. In this view, a subordinate is helping in return for an increase in its expected future reproductive success. However, it's also possible that subordinates help in return for immediate reproductive benefits. Helping may function as payment for a share of a parentage in a dominant's broods or for the opportunity to breed concurrently with dominants [22]. Finally, if subordinates are reproductively active then helping may be a reciprocal arrangement between dominants and subordinates, i.e., subordinates may help to raise dominants' broods in return for dominant assistance with the subordinates' own broods.acceptance in the group. Group membership confers access to group-held resources and increases a subordinate's likelihood of surviving long enough to obtain a breeding position in the future. In this view, a subordinate is helping in return for an increase in its expected future reproductive success. However, it's also possible that subordinates help in return for immediate reproductive benefits. Helping may function as payment for a share of a parentage in a dominant's broods or for the opportunity to breed concurrently with dominants [22]. Finally, if subordinates are reproductively active then helping may be a reciprocal arrangement between dominants and subordinates, i.e., subordinates may help to raise dominants' broods in return for dominant assistance with the subordinates' own broods.

Direct fitness effects of helping behaviour have not been tested in this species. For example, helpful subordinates might share parentage in dominants' broods or might breed concurrently with dominants. So instead of paying-to-stay for future benefits (e.g. increased survival, and either queuing for the breeding position or delaying dispersal until a nearby breeding vacancy becomes available), subordinates might be helpful for immediately acquired benefits, e.g. paying-to-reproduce. As long as the exact benefits from payment have not been measured, the functional reason(s) why subordinates perform ‘payments’ remain elusive (see also review by [22] concerning the various interpretations of the pay-to-stay hypothesis and the benefits subordinates might acquire from payment).

Here we report on experiments conducted with female cichlids to test whether immediate direct reproductive benefits accrued by subordinate females might explain their helping behaviour. For this purpose, only subordinate alloparental brood care of unrelated dominant females' broods, and dominant alloparental brood care for subordinate females' broods were considered [20], [21]. In nature both related and unrelated subordinates may associate with [5] and assist [8] the dominant pair. Individuals cannot gain inclusive fitness benefits from caring for unrelated broods, and therefore alloparental brood care can be seen as purely altruistic on a short term basis. Unrelated groups were created with either one subordinate female (treatment 1 from ref. 20), two subordinate females [21], or one subordinate female and one subordinate male [23]. We measured brood care, alloparental brood care and reproductive success of all female group members. We asked whether alloparental brood care is reciprocated, providing alloparental care benefits to the subordinates in the near future (payment as a form of reciprocity hypothesis [24]) Alternattively, if care is not reciprocated, subordinate alloparental care may be a form of payment to stay (pay-to-stay hypothesis). We then explicitly assess whether they pay for immediate reproductive benefits (pay-to-reproduce hypothesis) or whether benefits are not immediately acquired (suggesting they pay-for-future benefits). These hypotheses are not mutually exclusive, but merely distinguish what types of benefits subordinates might acquire from alloparental ‘payment’.

We address two questions. First, does helping function as payment for current reproductive opportunities? If helping behaviour plays this role then subordinates that perform alloparental brood care should be more likely to reproduce. Second, if subordinates are reproducing, then is subordinate alloparental brood care reciprocated by the dominant. The two benefits of helping behaviour are not mutually exclusive. For example, alloparental care by the subordinate might exceed that by the dominant but the two might nevertheless be positively correlated. This result would suggest that subordinate helping pays for both the opportunity to reproduce and some level of reciprocal helping by the dominant.

## Materials and Methods

We measured maternal and alloparental brood care and reproductive success (total number of eggs produced) of dominant and subordinate females in four different experiments, summing the data per female over 30 days. Combining the four data-sets was necessary to acquire a sufficiently large sample size of care observations and reproductive measurements.

### Experimental set-ups

We created artificial groups of three or four unrelated individuals. All fish were laboratory-reared descendants of fish caught at the southern end of Lake Tanganyika (near Mpulungu, Zambia). Fish were kept in large aggregation aquaria without access to breeding substrate prior to the experiment. All groups contained a breeder pair (large male and female). We measured the sizes of the fish at the start of each sequence of the experiments (body mass in mg and body size as standard length SL in mm), sexed them by examing the genital papilla, and marked all fish individually (by taking a small fin-clip from the dorsal and/or anal fin). Marking had no adverse effects on the fish. Body sizes SL mm±s.d. are reported throughout.

In the first experiment DH and IMH created 16 groups, each consisting of a dominant breeding pair and a subordinate female. These groups were concurrently used in another experiment; we selected only data from treatment 1 of that experiment [20], in which the breeding resources were closely spaced and where the territory of the dominant female encompassed all available breeding substrate. Treatment 2 was excluded from analyses because, in that treatment, the breeding resources were separated into two patches that were far apart. Subordinate females were then much more likely to defend one of those patches as a territory against the dominant female and to cease providing alloparental care. These females were as reproductively successful as the dominant females [20]. Body sizes of large helper females were 51.4±3.7 (*n* = 16). Data were collected over one sequence lasting 30 days. See [20] for more details, including the body measurements of the other group members.

In the second experiment, DH created 32 groups, each consisting of a dominant breeding pair and two subordinate females (one large and one small). Body sizes of large helper females were 46.3±5.4 (*n* = 32), small helper females 36.9±6.1 (*n* = 32). Data were collected over two sequences, each lasting 30 days. In between, breeder females were exchanged with new breeder females. Afterwards, the breeder females were removed and the large subordinate females gained the dominant breeding position; and also very small helper females were added of 31.2±5.3 mm SL (*n* = 32), to keep group size constant. Data were collected for another 30 days. See [21] for more details, including the body measurements of the other group members.

In the third experiment DH, EJ and JSM created 48 groups, of which 37 groups had at least one female subordinate. Of these 37 groups, 12 groups contained a large subordinate male (50.4±2.0 SL mm) and a small subordinate female (41.4±2.4 SL mm); 11 groups contained a large subordinate female (50.6±2.3 SL mm) and a small subordinate male (41.6±2.4 SL mm); 14 groups contained a large subordinate female (49.8±1.9 SL mm) and a small subordinate female (41.3±1.7 SL mm). In all four treatments, the dominant pair was always substantially larger than their subordinates. See [23] for more details, including the body measurements of the other group members.

In the fourth experiment DH created 16 groups containing a large subordinate female (44.7±3.4 SL mm). Data were collected over two sequences, the first lasting 30 to 87 days, the second 15 to 45 days. In between, the breeder pairs were exchanged between the different large subordinate females. At the start of the second sequence large subordinates were 50.7±4.3 SL mm. See D. Heg (in preparation) for more details, including the body measurements of the other group members.

In experiment 1, groups were maintained in separate compartments of a large semi-circular ringtank. The compartments housing the groups used in this paper (treatment 1 experiment 1), each contained four clay flowerpot halves close together (used as shelters and for breeding). In experiment 2 and 3, groups were maintained in adjacent 125 litre compartments within one 1000 litre aquarium. Compartments were separated by alternating clear and opaque partitions, such that each group could see one adjacent group from the same set of four. Compartments measured 65 cm length×32.5 cm breadth×65 cm height. The floor of the aquarium was covered with a layer of sand (ca. 6 cm). Each compartment contained: two clay flower pot halves, several translucent tubes (suspended near the surface, used as a refuge from aggression), and a suspended filter (also used as a refuge). The availability of refugia ensured that subordinates could always avoid interactions with dominants, who usually stayed near the pot halves. In experiment 4, groups were maintained in separate compartments of a large semi-circular ringtank, each compartment contained two flowerpot halves (see [15] for similar set-up).

After the body measurements were taken, the subordinates were released directly into their respective compartments. The dominant pair were kept overnight in single isolation nets within their compartments before being released. Water temperature was maintained between 25.0°C and 28.0°C. The light regime was a 13:11 h light:dark cycle. During experiment 1, 2 and 4 all groups were fed daily with commercial TetraMin flake food, supplemented with fresh food (*Artemia spp.*, *Daphnia spp.*, mosquito larvae) during two days per week, *ad libitum*. During experiment 3 all groups were fed TetraMin flake food only, 2.5% of the group's combined mass per day.

### Brood care observations

*N. pulcher* females clean the substrate of the breeding shelter starting one to two days before spawning and dig away excessive sand. During these days they also court dominant males intensively and may engage in ‘pseudo-spawning’ (behaviourally identical to spawning, but without eggs being laid). Spawning takes several hours and was recorded by direct observations and video-recording of the compartments. During spawning, subordinates are usually not allowed inside the breeding shelter by the dominants, but some exceptions occur. DNA microsatellite analysis from a sub-sample of broods from experiment 3 confirmed that we correctly identified the female who had spawned the eggs in 91 out of 91 dominant female broods and 7 out of 8 subordinate female broods [23]. In the one case where we failed to identify subordinate female spawning, DNA maternity analysis showed she had spawned simultaneously with the dominant female inside the same breeding shelter during the weekend, when observations were conducted less intensively. Therefore, for the final analyses of subordinate female reproduction, we assumed all broods were correctly assigned to their mothers based on the behavioural observations of pre-spawning courtship and actual spawning. Note that two females spawned simultaneously on the same day on only four occasions, three times (partly) inside the same breeding pot (including the case detected by DNA analyses), one time inside two separate pots.

After spawning was completed, maternal brood care (abbreviated ‘brood care’ throughout) and alloparental brood care (frequency of cleaning and fanning eggs combined) was determined for all group members simultaneously during a 15 min observation. Male brood care and alloparental brood care was rare and excluded from the analyses. Frequency of care was determined for 450 broods (Table 1). In the evening, clutches were removed and eggs counted (clutch size defined as the number of eggs surviving plus eaten).

**Table 1:** Sample sizes of brood care observations per mother.

	Potential alloparents					
Mother	D	L	S	L+S	D+L	D+S
Dominant **D**	-	202	22	167	-	-
Large subordinate **L**	30	-	0	-	-	23
Small subordinate **S**	1	0	-	-	6	-

- = not applicable

All experiments were approved by LANAT of the Kanton Bern, and thus complied with the legal requirements of Switzerland.

### Statistical analyses

Data analyses were conducted with SPSS16. Larger broods receive more care [23], therefore all brood care (maternal and alloparental care) was expressed as the proportion of the total brood care provided by all female group members. Reciprocity of alloparental brood care was analysed using Spearman's rank correlation, data summed over all clutches produced during one sequence. Note that subordinate alloparental care did not correlate with subordinate body size (SL), body mass (mg) or body condition ([body mass/SL3]*100; *n* = 265, Spearman's rank correlations, *r*_s_ = −0.08, *p* = 0.11; *r*_s_ = −0.10, *p* = 0.11; *r*_s_ = −0.09, *p* = 0.13, respectively). The total number of eggs produced per 30 days was related to the proportion of total brood care that consisted of subordinate alloparental brood care (i.e., subordinate brood care / total brood care by female group members, summed over all broods during one sequence), using GEE and a log-link, corrected for group effects, scaling parameter adjusted using the deviance method [25]. Female body size (the major determinant of female productivity [23]), was also included in the model. Coefficients for the parameters corrected for these effects are reported as *B* with their±standard error throughout.

## Results

Both dominant and subordinate females produced clutches. When both the dominants and subordinates produced clutches, dominants usually laid their first clutch before their subordinate(s) (average number of days since start sequence±s.d., all four experiments combined: dominants: 11.8±6.6 days, *n* = 44; large subordinates: 17.1±9.0 days, *n* = 36; small subordinates: 16.2±8.1, *n* = 13). Comparing within the group, dominant females were the first to produce a clutch in 34 out of 49 cases (average difference to subordinate females±s.d.: 5.0±10.2 days, one-sample *t*-test *t* = 3.42, *df* = 48, *p* = 0.001; in 2 cases dominant and subordinate female produced their first clutch on the same day). Therefore, although potentially reciprocal alloparental care could be initiated by the dominant or the subordinate, in the majority of cases subordinates could engage in alloparental care first, and dominants could react to this investment by adjusting their level of alloparental care accordingly.

Dominant females benefited when subordinates provided alloparental brood care, but the reverse was not true: dominant brood care was reduced when subordinates showed more alloparental brood care, but not vice versa (Table 2). Large subordinate females also reduced their level of brood care depending on the investment by small subordinate females, but again not vice versa (Table 2). Subordinates were also consistent in their level of alloparental care provided: there was a significant positive correlation between the proportion of alloparental care given in sequence t vs sequence t+1 (i.e. consistency of alloparental care comparing different broods from the same dominant female, Spearman *r*_s_ = 0.44, *n* = 98, *p*<0.001). Consistency in alloparental care was also detectable for the subset of subordinates who assisted a different dominant female in sequence t+1 (experiments 2 and 4: Spearman *r*_s_ = 0.37, *n* = 69, *p* = 0.002).

**Table 2:** Spearman rank correlations between the frequency of maternal brood care and frequency of alloparental care by other female group members, in brackets sample sizes (number of broods).

	Female alloparent		
Mother	Dominant	Large subordinate	Small subordinate
**Dominant**		−0.18 ** (369)	−0.30 *** (189)
**Large subordinate**	0.20 (53)		−0.48 * (23)
**Small subordinate**	−0.23 (7)	−0.13 (6)	

* *p* = 0.022;

** *p* = 0.001;

*** *p*<0.001.

**Figure 1:**
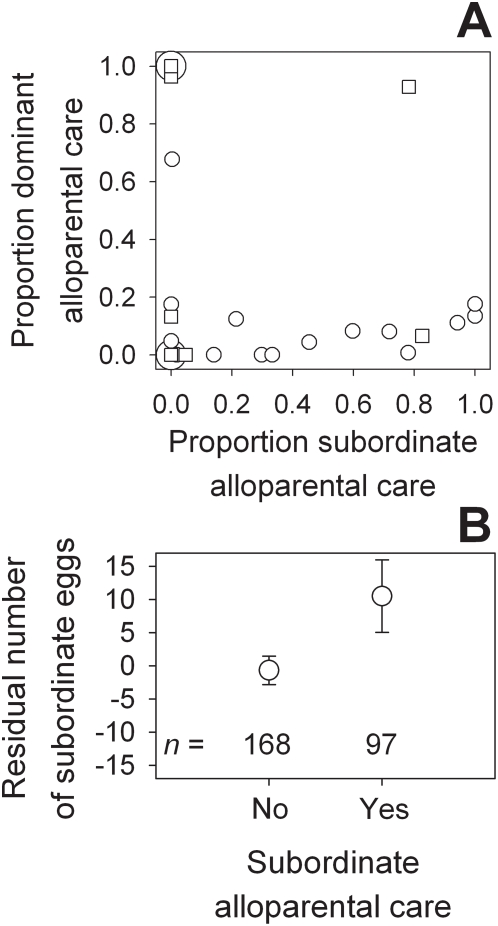
Reciprocity and subordinate female reproduction in *N. pulcher*. (A) No direct reciprocity in female cooperative cichlids: correlation between subordinate female alloparental care for dominant's broods and dominant female alloparental care for subordinate's broods (*n* = 26). Circles: large subordinate females vs dominant females (large symbol: two overlapping points); squares: small subordinate females vs dominant females. Proportion alloparental care is the alloparental care divided by the total care of all female group members, to correct for larger clutches receiving more care in general (see Materials and Methods and [23]). (B) Subordinate females produced more eggs when they provided alloparental brood care for the dominant females' broods. Depicted are the residual number of eggs produced per 30 days, corrected for the other fixed and random effects (see Table 3).

If direct reciprocity applies, there should be a positive correlation between the alloparental care given and the alloparental care received from the female group members. However, we found no evidence for direct reciprocity between subordinate alloparental brood care and dominant alloparental brood care (Figure 1a, Spearman *r*_s_ = −0.06, *n* = 26, *p* = 0.76). This result did not change when we selected the cases where the dominant had produced a brood first (Spearman *r*_s_ = 0.09, *n* = 22, *p* = 0.69), or when averages per group were used (Spearman *r*_s_ = −0.13, *n* = 21, *p* = 0.56). These results suggest that subordinate females do not pay because they can expect to get this payment reciprocated by the dominant females.

In contrast, we found clear evidence that subordinate females providing alloparental care gained benefits of increased direct reproduction (Fig. 1b, Table 3). Subordinate females that performed more alloparental care were more likely to produce eggs themselves. This effect was independent of subordinate body size, which also influenced reproduction positively (Table 3). We also detected significant differences between the experiments: female subordinate reproduction was more likely in experiments 1, 2 and 4 (one or two female subordinates), compared to experiment 3 (one female, one male subordinate, Table 3). Finally, subordinate reproduction did not depend on their size difference with the dominant female (Table 3).

**Table 3:** Reproductive output of subordinate female group members depending on their investment in alloparental care (proportion of total female care), their body size (SL mm), corrected for differences between the experiments (1, 2, 3 or 4).

	Total number of eggs / 30 days (*n* = 259)			
**Parameter**	χ^2^	*df*	*p*	*B*±SE
**Intercept**	1.8	1	0.17	−1.66±1.13
**Alloparental care**	6.0	1	0.014	1.49±0.69
**Body size SL**	13.7	1	<0.001	0.078±0.021
**Experiment**	7.5	3	0.059	experiment 1: 0.32±0.73
				experiment 2: −0.11±0.67
				experiment 3: −1.34±0.80
				experiment 4: 0 (reference)

GEE results with Wald χ^2^, degrees of freedom, *p*-values and coefficients *B*±s.e., corrected for group identity effects, and the scaling parameter adjusted using the deviance method. Total number of eggs / 30 days rounded to the nearest integer value. The difference in body size [dominant female - subordinate female] was non-significant at *p* = 0.75 and removed from the model.

## Discussion

Our results suggest that female subordinate cichlids pay with alloparental care to ensure that they can reproduce themselves (supporting the hypothesis that by payment they might acquire short-term reproductive benefits). The most likely mechanism is that by performing helping behaviour ensures that a subordinate has access to the breeding substrate, which she needs to lay eggs. This interpretation would also explain why subordinates compete for access to the breeding shelter, which might provide the best opportunities for both males and females to gain parentage [26]. Such results are likely not unique to this species; for example, female yellow-bellied marmots similarly adjust their social behaviour largely to get access to direct reproduction [27].

No evidence for ‘reciprocal altruism’ was found: i.e. there was no correlation between the amount of care that dominants provided for their subordinates' broods and subordinates' alloparental care for dominants' broods. Subordinate females on average also provided more alloparental care than did dominant females [21]. Evidence for ‘reciprocal altruism’ in animals is scarce [28]–[33], with some studies showing no evidence [32]. However, in many group-living vertebrates, subordinate reproduction is very limited, such that there is little opportunity for reciprocal alloparental care between dominants and subordinates [34]. In contrast, opportunities for reciprocation in *N. pulcher* are high, as female cichlids produce clutches about every second week and reproduction by subordinates is possible. ‘Delayed reciprocity’ may also be possible because of the existence of matrilines in nature (inheritance of the workforce [5]). Nevertheless, no evidence of reciprocity, at least at the short time scales used in this experiment, was found. We focused on small groups of unrelated individuals, in which choice of social partners was limited. In nature, within-group relatedness is highly variable. In addition, subordinate fish may move among groups and dominant fish may have several potential subordinate helpers from which to choose. It remains to be seen whether reciprocal alloparental brood care in cichlids may emerge in related dyads (e.g. matrilines); or appears when cichlids are free to chose the partners with whom they cooperate with [35]: the ‘biological market theory’ [36], [37].

Subordinate payment appears to be beneficial to the dominant female, since she is relieved of brood care duties, but not vice versa. Similarly, large subordinate females appear to benefit from alloparental care by small subordinate females [21]. Thus, although all females may show alloparental care for broods from the other female group members, females only downwardly adjust their workload in relation to the alloparental care provided by lower ranking females. It supports the notion that individuals pay ‘up’ the dominance hierarchy and is inconsistent with the idea of alloparental care being a case of egalitarian reciprocity.

The pay-to-stay hypothesis needs to be evaluated taking into account the immediate and future fitness stakes of the subordinates involved. A subordinate may pay-to-stay because staying increases survival [17], [38], [39], and therefore increases the likelihood of reaching a dominant, breeding position [18]. As we have shown, subordinates may be prepared to increase their payments in return for opportunities for current reproduction (the ‘pay-to-reproduce’ hypothesis). Finally, subordinates may adjust their level of help according to parentage, at least in females [20], [21], [23]. Field and laboratory studies indicate *N. pulcher* subordinate males may have some parentage in the brood [23], [40], [41] and female subordinates readily produce eggs, at least with unrelated dominant males [20], [21], [23]. Thus, a combination of immediate, near-term and long-term direct benefits may be accrued by subordinates that pay by providing help. It remains to be tested which physiological and ultimate factors cause within-subordinate variability in the propensity to provide alloparental care and thereby gain own reproduction. Subordinate females showed consistency in their level of alloparental care provided (both within- and between-dominant females assisted). This result supports the idea that female helpfulness is part of an aggressiveness-boldness-exploration continuum (‘behavioural syndrome’), where females of these various behavioural types might follow alternative life-history trajectories correlating with their propensity to provide help [42], [43].

The pay-to-stay hypothesis predicts that dominants punish or evict subordinates if these subordinates are not helpful or otherwise claim a larger share of reproduction not counterbalanced by any positive effects of the subordinates' presence. We focused on the relationship between helping behavior and receipt of alloparental care or reproductive opportunities. However, focusing on helping behaviour alone may be misleading, because conflicts may be resolved using other mechanisms. For example, in *N. pulcher*, subordinates may appease dominant breeders through submissive behaviours before they get evicted [16]. Submissiveness is an indicator of the subordinate's reproductive capacity, at least in male *N. pulcher* [44]. Indeed, the various experimental tests of the pay-to-stay hypothesis provide mixed results [15], [18], [45]. Measurements of the direct effects of helpfulness and other behavioral investments on eviction of subordinates, as well as more experimental manipulation of helpfulness [16] are needed. Such experiments would provide a thorough test of the pay-to-stay hypothesis.

In *N. pulcher* and in several other fish species, subordinates may make other costly adjustments, such as growth adjustments [19], [46], [47], to ensure continued group membership (‘strategic growth’). Subordinates reducing growth incur a cost due to a reduction in their capacity to lay eggs [20] or to produce sperm [48], [49] and gain parentage [23], but by doing so may reduce their costliness to more dominant group members. Since males incur direct fitness costs from shared parentage [23], whereas in females costs are lower or absent [20], [23], dominant males should be more sensitive to the number and sizes of subordinate males in their group than dominant females are to the number and sizes of subordinate females in their group. Consistent with these sex differences, ample evidence has now been accumulated that the dominant male grows faster than similar sized subordinate males and that the highest ranking subordinate male shows strategic adjustments in growth to ensure a safe-size difference with the dominant male and potentially prevent conflict [50] and eviction [15], [45], [51]. As expected, females do not show such adjustments, or considerably less pronounced adjustments than in the males [52]. In this study, our results show a negative correlation between the maternal brood care of higher ranking females and alloparental brood care of lower ranking females, suggesting there might be immediate benefits of more dominant females to accept smaller sized subdominant females as group members.

In conclusion, we did not find evidence of direct reciprocal ‘altruism’. However, we did find evidence of reciprocal benefits – help in exchange for opportunities to reproduce. This ‘pay-to-reproduce’ mechanism (with or without other pay-to-stay benefits) may be sufficient to explain helping behavior, at least in female subordinate cichlids.
